# Fine Particulate Air Pollution and the Progression of Carotid Intima-Medial Thickness: A Prospective Cohort Study from the Multi-Ethnic Study of Atherosclerosis and Air Pollution

**DOI:** 10.1371/journal.pmed.1001430

**Published:** 2013-04-23

**Authors:** Sara D. Adar, Lianne Sheppard, Sverre Vedal, Joseph F. Polak, Paul D. Sampson, Ana V. Diez Roux, Matthew Budoff, David R. Jacobs, R. Graham Barr, Karol Watson, Joel D. Kaufman

**Affiliations:** 1 Department of Epidemiology, University of Michigan, Ann Arbor, Michigan, United States of America; 2 Department of Environmental and Occupational Health Sciences, University of Washington, Seattle, Washington, United States of America; 3 Department of Biostatistics, University of Washington, Seattle, Washington, United States of America; 4 Department of Radiology, Tufts Medical Center, Boston, Massachusetts, United States of America; 5 Department of Statistics, University of Washington, Seattle, Washington, United States of America; 6 Los Angeles Biomedical Research Institute, Los Angeles, California, United States of America; 7 Division of Cardiology, University of California Los Angeles, Los Angeles, California, United States of America; 8 Division of Epidemiology and Community Health, University of Minnesota, Minneapolis, Minnesota, United States of America; 9 Departments of Medicine and Epidemiology, Columbia University Medical Center, New York, New York, United States of America; 10 Departments of Epidemiology and Medicine, University of Washington, Seattle, Washington, United States of America; University of Otago, New Zealand

## Abstract

**Background:**

Fine particulate matter (PM_2.5_) has been linked to cardiovascular disease, possibly via accelerated atherosclerosis. We examined associations between the progression of the intima-medial thickness (IMT) of the common carotid artery, as an indicator of atherosclerosis, and long-term PM_2.5_ concentrations in participants from the Multi-Ethnic Study of Atherosclerosis (MESA).

**Methods and Results:**

MESA, a prospective cohort study, enrolled 6,814 participants at the baseline exam (2000–2002), with 5,660 (83%) of those participants completing two ultrasound examinations between 2000 and 2005 (mean follow-up: 2.5 years). PM_2.5_ was estimated over the year preceding baseline and between ultrasounds using a spatio-temporal model. Cross-sectional and longitudinal associations were examined using mixed models adjusted for confounders including age, sex, race/ethnicity, smoking, and socio-economic indicators. Among 5,362 participants (5% of participants had missing data) with a mean annual progression of 14 µm/y, 2.5 µg/m^3^ higher levels of residential PM_2.5_ during the follow-up period were associated with 5.0 µm/y (95% CI 2.6 to 7.4 µm/y) greater IMT progressions among persons in the same metropolitan area. Although significant associations were not found with IMT progression without adjustment for metropolitan area (0.4 µm/y [95% CI −0.4 to 1.2 µm/y] per 2.5 µg/m^3^), all of the six areas showed positive associations. Greater reductions in PM_2.5_ over follow-up for a fixed baseline PM_2.5_ were also associated with slowed IMT progression (−2.8 µm/y [95% CI −1.6 to −3.9 µm/y] per 1 µg/m^3^ reduction). Study limitations include the use of a surrogate measure of atherosclerosis, some loss to follow-up, and the lack of estimates for air pollution concentrations prior to 1999.

**Conclusions:**

This early analysis from MESA suggests that higher long-term PM_2.5_ concentrations are associated with increased IMT progression and that greater reductions in PM_2.5_ are related to slower IMT progression. These findings, even over a relatively short follow-up period, add to the limited literature on air pollution and the progression of atherosclerotic processes in humans. If confirmed by future analyses of the full 10 years of follow-up in this cohort, these findings will help to explain associations between long-term PM_2.5_ concentrations and clinical cardiovascular events.

*Please see later in the article for the Editors' Summary*

## Abstract 2

In a prospective cohort study, Sara Adar and colleagues find that decreasing levels of fine particulate matter in multiple US urban areas are associated with slowed progression of intima-medial thickness, a surrogate measure of atherosclerosis.

## Editors' Summary

**Background:**

Cardiovascular disease (CVD)—disease that affects the heart and/or the blood vessels—is a major cause of illness and death worldwide. In the US, for example, the leading cause of death among adults is coronary artery disease, a CVD in which narrowing of the heart's arteries by atherosclerotic plaques (fatty deposits that build up with age inside arteries) slows the blood supply to the heart and may eventually cause a heart attack (myocardial infarction). The fourth leading cause of death in the US is stroke, a CVD in which atherosclerotic plaques interrupt the brain's blood supply. Smoking, high blood pressure, high blood cholesterol levels, diabetes, being overweight, and being physically inactive all increase an individual's risk of developing CVD. Treatments for CVD include lifestyle changes and taking drugs that lower blood pressure or blood cholesterol levels.

**Why Was This Study Done?:**

Another risk factor for CVD is long-term exposure to fine particulate air pollution. Fine particulate matter (PM_2.5_)—particles with a diameter of less than 2.5 µm or 1/30th the width of a human hair—is mainly produced by motor vehicles, power plants, and other combustion sources. Why PM_2.5_ increases CVD risk is unclear, but one hypothesis is that it initiates or accelerates atherosclerosis. In this prospective cohort study, which is part of the Multi-Ethnic Study of Atherosclerosis and Air Pollution (MESA Air), the researchers investigate whether there is an association between long-term PM_2.5_ exposure and the progression of intima-medial thickness (IMT; the tunica intima and media are the innermost layers of the arterial wall) in the right common carotid artery (one of the arteries that supplies the head and neck with blood). A prospective cohort study enrolls a group of individuals and follows them to see whether exposure to certain risk factors affects their risk of developing a specific disease; progression of IMT—thickening of the arterial wall with time—in the common carotid artery is a surrogate measure of atherosclerosis.

**What Did the Researchers Do and Find?:**

MESA Air enrolled nearly 7,000 45–84-year-old individuals without symptoms of CVD from different ethnic backgrounds living in six US metropolitan areas beginning in 2000. Carotid IMT was measured using ultrasound in all the participants at baseline and at the second or third planned examination of MESA Air for most participants. The researchers modeled the outdoor concentration of PM_2.5_ at each participant's house for the year preceding baseline and for the years between ultrasound examinations using data collected by regulatory monitoring stations and data derived from study-specific air samples collected outside the homes and in the communities of study participants. Among the 5,362 study participants eligible for the current analysis, the average annual progression of carotid IMT was 14 µm/year. Higher average levels of residential PM_2.5_ between ultrasound examinations were associated with increased IMT progression among people living in the same metropolitan area after adjusting for other factors that might have affected each participant's risk of CVD such as smoking and age. Living at a residence with 2.5 µg/m^3^ higher levels of residential PM_2.5_ between ultrasound examinations was associated with 5.0 µm/year greater IMT progression when compared to people living in less polluted regions of the same metropolitan area. Finally, after adjusting for PM_2.5_ levels at baseline, greater reductions in PM_2.5_ between ultrasound examinations were associated with a slower rate of IMT progression.

**What Do These Findings Mean?:**

These findings suggest that higher long-term PM_2.5_ concentrations are associated with increased IMT progression—a surrogate for atherosclerosis progression—and that larger reductions in PM_2.5_ are associated with slower IMT progression. By combining these findings with other results from MESA Air, the researchers estimate that individuals living in parts of town with 2.5 µg/m^3^ higher PM_2.5_ levels may have a 2% increased risk of stroke compared to people living in less polluted regions of the same metropolitan area. Because of study limitations such as the use of a surrogate marker for atherosclerosis and the failure to account for changes in other factors that might also have affected CVD risk, the findings reported here should be interpreted cautiously. Nevertheless, these findings support the hypothesis that long-term exposure to PM_2.5_ is associated with the progression of atherosclerosis and consequently with an increased risk of CVD, even at PM_2.5_ levels below existing regulatory standards.

**Additional Information:**

Please access these Web sites via the online version of this summary at http://dx.doi.org/10.1371/journal.pmed.1001430.

- The American Heart Association provides information for patients and caregivers on all aspects of cardiovascular disease (in several languages), including information on air pollution and cardiovascular disease
- The AIRNow site provides information about US air quality and about air pollution and health
- The Air Quality Archive has up-to-date information about air pollution in the UK and information about the health effects of air pollution
- The US Environmental Protection Agency has information on particulate matter pollution
- Information on MESA and on MESA Air is available

## Introduction

Long-term exposure to fine particulate air pollution (PM_2.5_) has been associated repeatedly with cardiovascular and ischemic heart disease [1]. Several biological processes underlying these associations have been proposed, including oxidative stress and systemic inflammation, endothelial dysfunction, and alterations in autonomic tone. Toxicological data also indicate that PM_2.5_ can initiate or accelerate atherosclerosis [2]–[6], yet there is little information to confirm this relation in humans.

Human investigations of prevalent atherosclerosis and air pollution have suggested a relation but are inconclusive. In cross-sectional analyses of older adults, 10 µg/m^3^ greater long-term concentrations of PM_2.5_ were associated with a 1%–10% larger intima-medial thickness of the common carotid artery (IMT) [7]–[9]. In young adults, a positive but non-significant association has also been reported [10]. Other atherosclerosis measures such as coronary artery calcium and ankle brachial index have been linked to traffic exposures [11],[12], a source of PM_2.5_, but have shown less consistent associations with PM_2.5_ itself [7]. Conversely, prevalent aortic calcium was linked to PM_2.5_ but not traffic [13]. Only one investigation to date has examined relations between air pollution and the progression of atherosclerosis in humans: both closer proximity to traffic sources and higher PM_2.5_ [14] were linked to greater progression of IMT in 1,483 older adults from Los Angeles. Replication of these findings in a more general population is needed, however, as participants of that study originated from five different clinical trials of vitamin, hormone, and anti-diabetes therapies and associations with IMT were limited to those in the treated groups. In addition, that investigation relied exclusively on regulatory monitoring data for assignment of air pollution concentrations and may have had insufficient information to fully capture fine-scale variability in pollution across different locations.

The Multi-Ethnic Study of Atherosclerosis and Air Pollution (MESA Air) was designed to investigate associations between long-term PM_2.5_ exposures and the progression of atherosclerosis over a 10-y follow-up period using information from the large population-based MESA cohort who were without pre-existing cardiovascular disease at baseline [15]. In this report, we present associations between individual-level PM_2.5_ estimated using measurements and models specific to this project and IMT progression over the first three MESA examinations. We hypothesized that persons living in areas with high PM_2.5_ during the follow-up period would experience a faster rate of progression than other individuals and that PM_2.5_ preceding the baseline exam would be related to baseline IMT.

## Methods

### Study Population

Participants of MESA with any IMT measurements in the first three clinical visits (2000–2005) who consented to having their home addresses geocoded were examined. While IMT measurements were also collected in later clinical visits of the MESA study, these data were collected from a different portion of the vessel and must be explored separately. At baseline, MESA was composed of 6,814 white, African-American or black, Spanish/Hispanic/Latino, and Chinese adults (aged 45–84 y) without clinical cardiovascular disease from six US communities (Baltimore, MD; Chicago, IL; Forsyth County, NC; Los Angeles County, CA; Northern Manhattan and Southern Bronx, NY; and St Paul, MN) [16]. Each field center developed recruitment procedures according to the characteristics of its community, past experience, available resources, and site-specific logistics. Recruitment sources included lists from county assessors, the Department of Motor Vehicles, local labor unions, commercial mailers, and random digit dialing. Friends, family, and persons serviced by the Centers for Medicare and Medicaid Services were also contacted to facilitate the target recruitment in upper age groups. This study met with the guidelines of the Declaration of Helsinki. Institutional review board approval was granted at each study site and written informed consent was obtained from all participants. We restricted our primary analyses to participants with complete covariate information.

### Common Carotid IMT

Trained technicians captured images of the right common carotid artery from supine participants using high resolution B-mode ultrasound (Logiq 700, 13MHz; GE Medical Systems). Images collected over a distance 10 mm proximal to the common carotid bulb were transferred from each study center to the Tufts Medical Center for quantification [16]. This analysis examined the mean far wall thickness of the right common carotid, retrospectively gated to end-diastole. Blinded replicate readings gave inter-reader intra-class correlation coefficients of 0.84 and 0.86 for two separate sets of readers [17]. IMT was collected from all participants at baseline with follow-up measures collected on a subset in exam 2 and a different subset in exam 3.

### Participant Characteristics

Information regarding participant demographics, medical history, and medications were obtained at each MESA exam through interviewer-administered questionnaires. Race/ethnicity was assessed by participant questionnaire where they were asked to report if they were best described as African-American or black, Asian (Chinese, Filipino, Japanese, Korean, Vietnamese, Asian Indian), white, Native Hawaiian or other Pacific Islander (Guamanian or Chamorro, Samoan, Micronesian, Tahitian), or American Indian or Alaska Native. Participants were also asked if they described themselves as Spanish/Hispanic/Latino and they were permitted to select more than one group. Participants reporting African-American or black, white, Spanish/Hispanic/Latino, or Chinese were eligible for participation. Measurements of anthropometry as well as serum levels of high-density lipoprotein (HDL) and low-density lipoprotein (LDL) cholesterol, glucose, homocysteine, and inflammatory markers were also collected [16]. Residential addresses were gathered and assigned geographic coordinates using ArcGIS v9.1 (ESRI) on the basis of the Dynamap 2000 street network (TeleAtlas).

### Air Pollution Concentrations

Individual-level, long-term PM_2.5_ concentrations were estimated by MESA Air for the MESA cohort using area-specific hierarchical spatio-temporal models described elsewhere [18],[19]. Predictions were derived from 2-wk average PM_2.5_ concentrations from the Environmental Protection Agency's Air Quality System (AQS) and supplemental monitoring specific to MESA Air [20]. These models decompose the space-time field of concentrations into spatially varying long-term averages, spatially varying seasonal and long-term trends, and spatially correlated but temporally independent residuals. Each model utilized spatial covariates such as proximity to roadways and local land uses to predict outdoor concentrations at subjects' homes between 1999 and 2007. City-specific cross-validated root mean square errors for these predictions ranged between 4.7% and 9.5% of long-term average concentrations at MESA Air monitoring locations.

Historical exposures accounting for residential history were estimated for each participant on the basis of concentrations for the year preceding their baseline exam. Exposure between ultrasounds was estimated by taking the time-weighted average of concentrations at a participant's residence or residences for the period between baseline and the follow-up exam. We also explored associations of IMT progression with the difference in exposures between the follow-up period and baseline levels as well as with average concentrations of PM_2.5_ measured at the nearest AQS monitor over the year before baseline and with living near a major roadway (i.e., within 100 m of an interstate or US highway or within 50 m of a state or county highway as defined by the US Census Feature Class Codes A1, A2, and A3).

### Statistical Analysis

A longitudinal mixed model [21] was fit with random slopes and intercepts for each subject in R v.2.10.1 [22]. As discussed in detail in Text S1, this model simultaneously examined the association between IMT at baseline and PM_2.5_ levels preceding the baseline exam (henceforth referred to as “cross-sectional association”) as well as IMT progression as a function of the average concentration over follow-up (henceforth referred to as “longitudinal association”). We also fit the same model examining cross-sectional and longitudinal associations and baseline PM_2.5_ as well as longitudinal associations with the change in PM_2.5_ between follow-up and baseline, defined as the average PM_2.5_ over follow-up minus baseline PM_2.5_. This specification allowed us to independently assess the associations of IMT progression with each of these two distinct exposures.

All models were constructed in a staged manner to assess the sensitivity of our results to control for different risk factors, including some that may possibly be mediators of the association between air pollution and IMT. In our minimally adjusted models, we explored confounding by age, sex, and race/ethnicity. Our moderately adjusted models added control for education, a neighborhood socio-economic score (derived from census tract level data on education, occupation, median home values, and median household income) [23], adiposity (1/height, 1/height^2^, weight, waist, and 1/hip), and pack-years at baseline as well as a time-varying smoking status. Our main models further adjusted for time-varying HDL, total cholesterol, statin use, diabetes mellitus (using the 2003 ADA fasting criteria algorithm [24]), systolic blood pressure, diastolic blood pressure, hypertensive diagnosis, and hypertensive medications. For sensitivity analyses, we tested an extended model that also included physical activity, alcohol use, second-hand smoke exposures, C-reactive protein, creatinine, fibrinogen, occupation, and neighborhood noise. Although we also considered changes in healthy food stores over the follow-up period from the National Establishment Time Series database (Walls & Associates) as an indicator of neighborhood change, it was uncorrelated with changes in pollution (ρ: −0.03 to −0.13) and thus not included in our models. All covariates were included as possible confounders of associations of PM_2.5_ with baseline IMT (cross-sectional associations) and with progression in IMT (longitudinal associations). Since metropolitan area was also considered to be an important potential confounder, models were constructed with and without fixed effects for clinic.

Although our models control for baseline IMT by including key predictors of IMT at baseline, we also explored a model of the change in IMT between the baseline and follow-up exams as the outcome normalized by the time between visits. Effect modification was also examined by age, gender, race/ethnicity, education, obesity, diabetes, hypertension, statin therapy, and baseline IMT. Standard model diagnostics were explored including graphics of residuals for evidence of non-normality, influential outliers, and omitted covariates.

Results were reported scaled to inter-quartile range of 2.5 and 1 µg/m^3^ for PM_2.5_ and the change in PM_2.5_ over the follow-up period, respectively. To illustrate our findings graphically, we present mean IMT levels and confidence intervals around those means for follow-up concentrations 3, 5, and 7 µg/m^3^ larger than the city average.

## Results

Between exams 1 and 3, 11,270 valid IMT measurements were collected from 5,660 participants. By design, approximately half of the participants were sampled in exam 2 (*n* = 2,907) and half in exam 3 (*n* = 2,726), with 99% contributing two samples per person. Excluding 645 observations with missing covariate information, and 513 with missing exposures resulted in 10,220 observations for this analysis. The 5,362 included participants (52% female) had a mean age of 62 y and were 40% white, 27% black, 21% Hispanic, and 12% Chinese (Table 1). Overall, 44% had hypertension, 12% had diabetes, and nearly 50% were former or current smokers at baseline. Some differences were observed between the MESA clinics with respect to race, ethnicity, and socio-economic features. New York and Los Angeles had a higher fraction of their populations without high school educations whereas Baltimore, Chicago, and Winston-Salem had larger fractions of participants with graduate degrees.

**Table 1:** Study population characteristics presented as mean (standard deviation) or percent.

Characteristics	Overall	Winston Salem	New York	Baltimore	St Paul	Chicago	Los Angeles
**Number of samples**							
Baseline	5,276	856	835	734	847	986	1,018
Follow-up	4,944	797	795	690	777	948	937
**IMT**							
Baseline (µm)	678 (189)	725 (207)	677 (173)	695 (191)	641 (165)	647 (180)	690 (199)
Progression (µm/y)	14 (53)	13 (56)	9 (50)	19 (65)	15 (49)	19 (55)	12 (43)
Follow-up time (y)	2.5 (0.8)	2.4 (0.8)	2.6 (0.7)	2.4 (0.8)	2.4 (0.9)	2.3 (0.8)	2.5 (0.9)
**Air pollution concentrations**							
Baseline PM_2.5_ (µg/m^3^)	16.6 (3.7)	15.5 (0.7)	15.5 (0.8)	15.2 (0.9)	11.9 (1.1)	16.9 (1.2)	23 (1.9)
Average follow-up PM_2.5_ (µg/m^3^)	15.5 (3.5)	14.5 (0.7)	15.0 (0.7)	14.9 (0.8)	10.4 (0.7)	15.5 (1.1)	21.4 (1.8)
Delta PM_2.5_ (µg/m^3^)	−1.1 (1.1)	−1.1 (0.4)	−0.5 (0.4)	−0.3 (0.5)	−1.4 (0.9)	−1.4 (0.8)	−1.6 (1.9)
**Personal characteristics**							
Age (y)	62 (10)	62 (10)	62 (10)	63 (10)	60 (10)	62 (10)	63 (11)
Female (%)	52	53	55	52	50	54	50
Race/ethnicity (%)							
White	40	53	20	51	60	49	12
Black	27	47	33	49	0	25	12
Chinese	12	0	0	0	0	26	38
Hispanic	21	0	47	0	40	0	39
Education (%)							
Less than high school	16	7	25	10	16	7	31
High school	18	22	18	19	22	8	19
Higher education	47	52	41	48	51	49	41
Advanced degree	19	19	16	22	11	36	9
Smoking status (%)							
Never	51	45	52	47	44	52	63
Former	37	43	34	40	41	37	28
Current	12	13	14	12	15	11	9
**General health characteristics**							
Body mass index (kg/m^2^)	28.2 (5.3)	28.7 (5.2)	28.7 (5.3)	29.3 (5.6)	29.4 (5.1)	26.7 (5)	27 (5.2)
Systolic BP (mm Hg)	126 (21)	133 (21)	125 (21)	128 (21)	122 (20)	123 (21)	126 (22)
Diastolic BP (mm Hg)	72 (10)	74 (10)	73 (10)	72 (10)	70 (10)	71 (10)	71 (10)
HDL (mg/dl)	51 (15)	51 (15)	53 (15)	52 (15)	49 (14)	54 (16)	49 (14)
LDL (mg/dl)	117 (31)	114 (30)	118 (32)	118 (31)	121 (31)	117 (31)	117 (31)
CRP (mg/dl)	3.7 (5.6)	4.4 (6.6)	3.4 (4.2)	4.0 (5.7)	3.9 (5.5)	3.1 (5.7)	3.3 (5.4)
Hypertension (%)	44	54	47	50	34	37	42
Statin users (%)	15	16	16	19	12	15	13
Diabetes (%)	12	11	13	13	10	8	15

Personal characteristics as reported at baseline. 86 participants had follow-up IMT measurements without valid baseline IMT measurements. Hypertension was defined by diastolic blood pressure ≥90, a systolic blood pressure ≥140 or self-reported history of hypertension with use of hypertensive medications.

CRP, C-reactive protein.

Among the whole population, we observed a mean baseline IMT of 678 µm and progression of 14 µm/y over a mean follow-up of 2.5 y. The mean long-term PM_2.5_ concentration was 16.6 µg/m^3^±3.7 µg/m^3^ with a range of 9.4 to 27.5 µg/m^3^. Concentrations were substantially more variable across areas (standard deviation: 3.5 µg/m^3^) than within areas (average standard deviation: 1.11µg/m^3^). As shown in Table 1, all areas exhibited a decrease in PM_2.5_ concentrations over the follow-up period (mean change: −1.1±1.1 µg/m^3^) but regions with higher baseline PM_2.5_ concentrations experienced the largest reductions over the follow-up period (overall ρ for baseline and change in PM_2.5_: −0.32 and average within-area ρ: −0.54). Concentrations at regulatory monitors also demonstrated similar patterns. While Chicago and New York had large fractions of their cohort living near major roadways (30% and 57%, respectively), in the other areas approximately 20% of the cohort resided in close proximity to a major roadway. Virtually no participants (<0.1%) changed residential proximity to roadways over follow-up.

**Figure 1:**
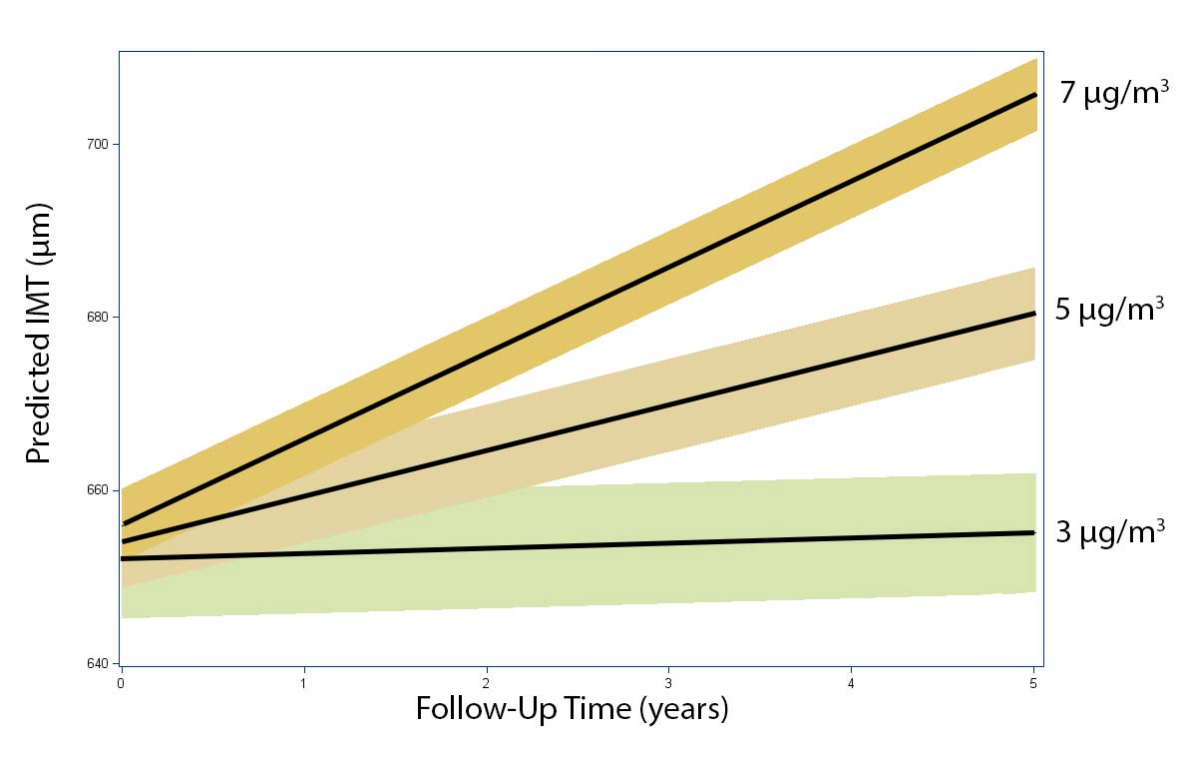
Estimated IMT (95% CIs) over time at varying levels of average residential PM_2.5_ concentrations exceeding the city average during the follow-up period. IMT estimated from results reported in Table 1 assuming a group of white women of average age, body mass index, LDL cholesterol, systolic and diastolic blood pressure, C-reactive protein, glucose, and baseline exposures to air pollution who never smoked, were not on hypertensive medications, and were in the lowest income and education groups. Results are reported for concentration increments above the city mean with confidence intervals around the mean.

Average PM_2.5_ concentrations over follow-up showed consistent positive associations with IMT progression in all models following adjustment for metropolitan area, with areas of higher concentrations showing steeper progressions of IMT over time (Figure 1). Living at a residence with a 2.5 µg/m^3^ higher concentration (inter-quartile range [IQR]) during the follow-up period was associated with a 5.0 µm/y (95% CI 2.6 to 7.4 µm/y) faster change in IMT over time when compared to others in the same metropolitan area (Table 2). Models that simultaneously explored associations with baseline PM_2.5_ and the change in PM_2.5_ over the follow-up period similarly indicated that a 2.5 µg/m^3^ larger baseline PM_2.5_ was associated with a 3.8 µm/y (95% CI 1.2 to 6.4 µm/y) faster rate of progression among persons with the same change since baseline. In addition, a 1 µg/m^3^ greater reduction in PM_2.5_ over follow-up was associated with a 2.8 µm/y (95% CI 1.6 to 3.9 µm/y) slower rate of IMT progression (Table 3) after control for metropolitan area and concentration preceding the baseline exam.

**Table 2:** Mean differences (95% CI) in IMT at baseline and in IMT progression over time associated with PM_2.5_ concentrations prior to baseline and averaged over follow-up, with and without control for metropolitan area.

Model	Overall Associations	Within-City Associations
**Baseline IMT (µm) per 2.5 µg/m^3^ of baseline PM_2.5_**		
Minimal adjustment	6.1 (2.6 to 9.6)	3.3 (−5.9 to 12.5)
Moderate adjustment	6.6 (3.1 to 10.2)	1.0 (−8.6 to 10.5)
Main model	6.3 (2.8 to 9.8)	0.4 (−9.1 to 9.9)
Extended adjustment	5.7 (1.5 to 9.8)	1.1 (−9.8 to 12.0)
**Progression of IMT (µm/y) per 2.5 µg/m^3^ of average follow-up PM_2.5_**		
Minimal adjustment	0.4 (−0.4 to 1.2)	4.8 (2.4 to 7.1)
Moderate adjustment	0.5 (−0.3 to 1.3)	4.9 (2.5 to 7.3)
Main model	0.4 (−0.4 to 1.2)	5.0 (2.6 to 7.4)
Extended adjustment	0.5 (−0.4 to 1.5)	4.4 (1.6 to 7.3)

Minimal adjustment included age, sex, and race/ethnicity. Moderately adjustment added control for education, a neighborhood socio-economic score (derived from census tract level data on education, occupation, median home values, and median household income), adiposity (1/height, 1/height^2^, weight, waist, and 1/hip), and pack-years at baseline as well as a time-varying smoking status. Main models further adjusted for HDL, total cholesterol, statin use, diabetes mellitus (using the 2003 ADA fasting criteria algorithm), systolic blood pressure, diastolic blood pressure, hypertensive diagnosis, and hypertensive medications. In sensitivity analyses, we tested an extended model that also included physical activity, second-hand smoke exposures, alcohol use, C-reactive protein, creatinine, fibrinogen, occupation, and neighborhood noise among a smaller subset of the population with complete data.

**Table 3:** Mean differences (95% CI) in IMT at baseline and in IMT progression over time associated with PM_2.5_ concentrations prior to baseline and change between follow-up and baseline, with and without control for metropolitan area.

Model	Overall Associations	Within-City Associations
**Mean IMT (µm) per 2.5 µg/m^3^ of baseline PM_2.5_**		
Minimal adjustment	6.4 (2.9 to 9.9)	5.4 (−4.0 to 14.7)
Moderate adjustment	7.0 (3.4 to 10.5)	3.3 (−6.5 to 13.0)
Main model	6.7 (3.2 to 10.2)	2.7 (−6.9 to 12.4)
Extended adjustment	6.0 (1.8 to 10.1)	3.2 (−8.0 to 14.3)
**IMT progression (µm) per 2.5 µg/m^3^ of baseline PM_2.5_**		
Minimal adjustment	0.3 (−0.5 to 1.1)	3.7 (1.3 to 6.2)
Moderate adjustment	0.3 (−0.5 to 1.1)	3.7 (1.1 to 6.3)
Main model	0.3 (−0.6 to 1.1)	3.8 (1.2 to 6.4)
Extended adjustment	0.4 (−0.5 to 1.4)	3.5 (0.5 to 6.5)
**IMT progression (µm) per 1 µg/m^3^ of change in PM_2.5_ over follow-up**		
Minimal adjustment	1.1 (0.2 to 2.0)	2.7 (1.6 to 3.8)
Moderate adjustment	1.2 (0.3 to 2.1)	2.7 (1.6 to 3.9)
Main model	1.3 (0.4 to 2.2)	2.8 (1.6 to 3.9)
Extended adjustment	1.0 (−0.1 to 2.0)	2.5 (1.1 to 3.9)

Change was defined as the average concentration over the follow-up period: concentration at baseline such that a reduction in concentrations over time would have a negative change and increases in concentrations over time would be manifest as a positive change. Minimal adjustment included age, sex, and race/ethnicity. Moderately adjustment added control for education, a neighborhood socio-economic score (derived from census tract level data on education, occupation, median home values, and median household income), adiposity (1/height, 1/height^2^, weight, waist, and 1/hip), and pack-years at baseline as well as a time-varying smoking status. Main models further adjusted for HDL, total cholesterol, statin use, diabetes mellitus (using the 2003 ADA fasting criteria algorithm), systolic blood pressure, diastolic blood pressure, hypertensive diagnosis, and hypertensive medications. In sensitivity analyses, we tested an extended model that also included physical activity, alcohol use, second-hand smoke exposures, C-reactive protein, creatinine, fibrinogen, occupation, and neighborhood noise among a smaller subset of the population with complete information.

**Figure 2:**
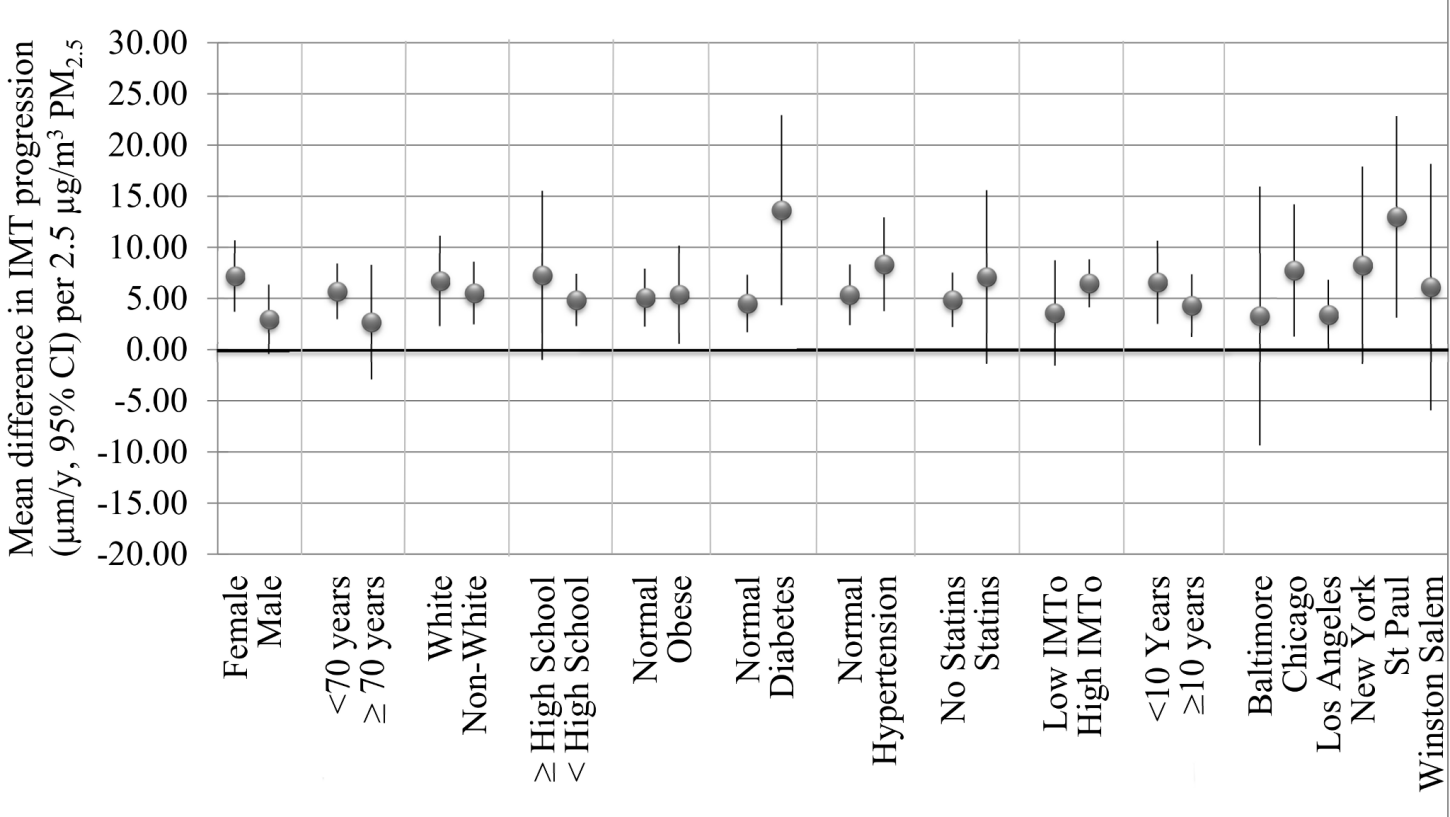
Mean difference in IMT progression (µm/y, 95% CI) per 2.5 µg/m^3^ PM_2.5_ concentration averaged over follow-up in select stratified analyses controlled for metropolitan area. Models controlled for age, sex, race/ethnicity, education, a neighborhood socio-economic score (derived from census tract level data on education, occupation, median home values, and median household income), adiposity (1/height, 1/height^2^, weight, waist, and 1/hip), pack-years at baseline, smoking status, HDL, total cholesterol, statin use, diabetes mellitus (using the 2003 ADA fasting criteria algorithm), systolic blood pressure, diastolic blood pressure, hypertensive diagnosis, hypertensive medications, and metropolitan area.

Without control for metropolitan area, associations between IMT progression and the average PM_2.5_ concentration over follow-up (0.4 µm/y [95% CI −0.4 to 1.2 µm/y per 2.5 µg/m^3^]) were positive but could not be distinguished from no association. The same was true for associations between progression and baseline PM_2.5_ in models controlled for the change in pollution over follow-up (0.3 µm/y [95% CI −0.6 to 1.1 µm/y] per 2.5 µg/m^3^). In all of the six metropolitan areas, however, increased IMT progression was observed with larger PM_2.5_ concentrations (Figure 2). Also, the change in PM_2.5_ over the follow-up period remained associated with IMT progression (1.3 µm/y reduction [95% CI 0.4 to 2.2 µm/y] per µg/m^3^ reduction in PM_2.5_), even without control for metropolitan area.

Cross-sectional associations with baseline IMT could not be differentiated from no effect (0.4 µm [95% CI −9.1 to 9.9 µm] [Table 2] to 2.7 µm [95% CI −6.9 to 12.4 µm] [Table 3]) after adjustment for the mean concentration for each metropolitan area. Associations with baseline IMT were stronger without control for metropolitan area. In these models, a 2.5 µg/m^3^ higher baseline PM_2.5_ concentration was associated with a 6.3 µm (95% CI 2.8–9.8 µm [Table 2]) to 6.7 µm (95% CI 3.2–10.2 µm, [Table 3]) larger baseline IMT.

Sensitivity analyses demonstrated that our findings were robust to increasing degree of control for an expanded covariate list (extended adjustment, Tables 2 and 3), restriction to residentially stable participants (no moves within 10 y, Figure 2), and alternate modeling strategies including a model of the change in IMT over the follow-up period as a function of the change in PM_2.5_ (see Text S1). Furthermore, qualitatively similar associations for baseline IMT and IMT progression were found for concentrations estimated by the nearest regulatory monitor. Living near a major roadway was not associated with a smaller baseline IMT or progression of IMT (Text S1).

Associations between PM_2.5_ and IMT and progression generally showed very little difference by risk factors examined, though stronger associations were suggested for some subgroups including women, diabetics, hypertensives, and residents of St Paul (Figure 2).

## Discussion

In a large prospective cohort study of adults without pre-existing cardiovascular disease, we found evidence that individuals with higher long-term residential concentrations of PM_2.5_ experience a faster rate of IMT progression as compared to other people within the same metropolitan area. Improvements in air quality over the duration of the study were similarly associated with changes in IMT progression, with greater reductions in PM_2.5_ showing slower IMT progression. These findings suggest that higher long-term PM_2.5_ exposures may be associated with an acceleration of vascular pathologies over time. As such, they may help explain why epidemiological studies have repeatedly found much larger associations between mortality and chronic air pollution exposures than can be explained by short-term triggering of cardiovascular events alone. Our findings furthermore bolster recent reports that falling pollution levels in the United States after the adoption of the Clean Air Act are associated with reduced mortality [25] and increased life expectancy [26],[27].

Our results indicate that persons living in residences with a 2.5 µg/m^3^ greater PM_2.5_ concentration could experience a 5.0 µm/y (95% CI 2.6–7.4 µm/y) faster rate of IMT progression than other persons in the same city. Similarly, a person who experienced a 1 µg/m^3^ larger reduction in PM_2.5_ over the follow-up period would have a 2.8 µm/y (95% CI 1.6–3.9 µm/y) slower IMT progression than another in the same city with the same baseline PM_2.5_. Although a recent meta-analysis [28] raises some questions as to the exact clinical implications of a larger IMT progression, results from the MESA cohort [17] suggest that participants living in parts of town with 2.5 µg/m^3^ higher concentrations of PM_2.5_ would have a 2% relative increase risk in stroke as compared to persons in a less polluted part of the metropolitan area. These findings have practical relevance since associations with IMT progression were found at concentrations commonly occurring in developed nations and well below those in developing countries. Although our mean long-term concentrations (range 10–23 µg/m^3^) were slightly above the new annual average US National Ambient Air Quality Standard of 12 µg/m^3^ and the World Health Organization guideline of 10 µg/m^3^, our findings are expected to hold even at lower concentrations as past evidence suggests that there is likely no safe threshold for air pollution [29].

The acceleration of atherosclerosis has been proposed as a possible mechanism linking chronic exposures to air pollution to clinical cardiovascular disease [30]–32; yet this is only the second publication to investigate the longitudinal relationships between air pollution and a surrogate of atherosclerosis in humans. Our findings support the hypothesis proposed by Künzli and colleagues [33] that persons living in areas with higher long-term concentrations of PM_2.5_ may experience a more rapid development of vascular pathologies, which leads to the development of clinically relevant atherosclerosis at an earlier age, and increases the population at risk of cardiovascular events. Our findings that concentrations preceding baseline had slightly weaker associations with IMT progression per unit change than those during the follow-up period may indicate the importance of recent exposures or reduced exposure measurement error during the study period.

The magnitude of our findings are consistent with Künzli et al., which reported a 0.6 µm/y (95% CI −0.1 to 1.4 µm/y) larger IMT progression per 2.5 µg/m^3^ of PM_2.5_ and a 5.5 µm/y (95% CI 0.1–10.8 µm/y) larger progression for living within close proximity to a major roadway [14]. While we observed larger PM_2.5_ associations, the 1,483 adult participants of that collection of studies were slightly younger, more white and Hispanic, better educated, and with lower overall rates of progression than our cohort. In addition, that study used a different exposure prediction modeling approach and relied on far fewer air pollution monitors than were available to us, resulting in nearly 5 times less variable PM_2.5_ estimates for Los Angeles than in this investigation. Nevertheless, their PM_2.5_ association was well within our confidence intervals for MESA participants in Los Angeles (3.4 µm/y; 95% CI −0.002 to 6.8 µm/y per 2.5 µg/m^3^). Toxicological data also support our findings, with several studies documenting the growth of atherosclerotic lesions in the coronary arteries and aortas of rabbits and mice following controlled exposures to particulate matter. [2]–[4],[34].

We also demonstrated positive cross-sectional associations between baseline IMT and long-term exposure but these were blunted and could not be distinguished from no association after control for metropolitan area. Associations similar to our between-city results have been previously reported for long-term exposure to PM_2.5_ among the older adults enrolled in the Los Angeles clinical trials [8], an earlier investigation of the MESA cohort at baseline [7], and a large population-based cohort of German older adults [9]. In fact, our result of a 3–10 µm difference in IMT at baseline is very consistent with the range of 5 to 17 µm predicted by these other studies for the same unit change in PM_2.5_ and slightly higher than a recent investigation of young adults that reported a 2 µm larger IMT predicted per 2.5 µg/m^3^ [10]. Associations between air pollution and other indicators of atherosclerosis extent have been somewhat suggestive but inconsistent [7],[11]–[13]. Since our cross-sectional results were driven by differences in baseline IMT between the two areas with the highest (Los Angeles) and lowest (St Paul) concentrations of PM_2.5_, however, and were not robust to control for metropolitan area, there is the possibility of residual confounding by regional factors.

In contrast to our cross-sectional results for baseline IMT, associations with IMT progression were strongest after control for metropolitan area. The reasons for the opposite effect of site adjustment on associations with baseline IMT and IMT progression remain to be determined. Because cross-sectional associations with baseline IMT are based on between-person contrasts, these relations may be more affected by confounding by personal factors than those in our progression models, which leverage information from the same individual. Within-area associations for IMT progression showed little change with control for neighborhood socio-economic characteristics, personal education, and perceived noise and demonstrated positive associations across all six metropolitan areas in stratified analyses. Changes in concentrations over the follow-up period were also associated with IMT progression in models with and without control for metropolitan area. Thus, while some questions are raised as to the robustness of cross-sectional associations with baseline IMT, sensitivity analyses raise our confidence in the associations with IMT progression as potentially reflecting a causal association.

These data come from a well-defined prospective cohort study with an uncommonly rich set of air pollution measurements in participants' communities and homes, including individual-level perceived noise exposures. The inclusion of noise data is a unique feature of this analysis as noise has generally not been accounted for in American epidemiological studies of air pollution to date. Although noise has been independently associated with cardiovascular disease and perceived noise was related to air pollution concentrations in MESA [35],[36], interestingly, we found no evidence of confounding of the relationship between air pollution and IMT progression by perceived noise in this analysis.

Despite the many strengths of this study, this work is not without its weaknesses. First, IMT likely does not capture all of the relevant pathophysiology related to air pollution exposures [37]. Second, our exposure assignment is currently limited to predictions of pollution from ambient origin after 1999 but restriction of the analysis to non-movers (≥10 y at baseline address) did not alter our findings. Third, we did not achieve complete follow-up of all participants and data. The probability of being lost to follow-up over these first three exams was unrelated to baseline IMT levels, however, and the likelihood of missing covariate or exposure data was also unrelated to baseline IMT or IMT progression. Missing covariate information was similarly unrelated to baseline exposure concentrations. This finding suggests that bias in our primary associations due to selection is unlikely although it is always a possibility in any longitudinal study. Furthermore, we are currently not accounting for changes in neighborhood characteristics that also may have occurred during the study period. Control for time-varying vascular risk factors in our extended adjustment model, which may capture some time-varying socio-economic trends, did not substantially alter our findings so we might hypothesize that this is not a major source of confounding. The lack of an association between reductions in air pollution and changes in healthy food stores is further supportive of this hypothesis. Nevertheless, future work through MESA will address this question more thoroughly as they explore the impacts of changing neighborhoods on health. Similarly, our exposure assessment does not currently account for the penetration of outdoor particles into indoor air but correlations of outdoor and indoor PM_2.5_ of outdoor origin have been shown to be high [38]. Future analyses of MESA Air will confirm the findings of this early dataset using IMT data collected during MESA clinical visits 4 and 5. These analyses will furthermore incorporate estimates of air pollution infiltration into participant homes and participant time-activity information, as well as investigate other correlated pollutants that may explain some of this PM_2.5_ association and explore relationships with clinical events.

Overall, these results for IMT in the first three exams of a large, multi-center, population-based cohort study support the hypothesis that PM_2.5_ may be associated with the progression of atherosclerosis, even at levels below existing regulatory standards. Such a pathway would lend further support to reported associations between air pollution and the incidence of clinical cardiovascular disease.

## Supporting Information

Text S1 **Extended methods and results.**
(DOCX)

## Abbreviations

HDL: high-density lipoprotein
IMT: intima-medial thickness
LDL: low-density lipoprotein
MESA: Multi-Ethnic Study of Atherosclerosis; Multi-Ethnic Study of Atherosclerosis and Air Pollution (MESA Air)
PM_2.5_: fine particulate matter.
