# Supplementary material for: Fine Particulate Air Pollution and the Progression of Carotid Intima-Medial Thickness: A Prospective Cohort Study from the Multi-Ethnic Study of Atherosclerosis and Air Pollution
Source: PLoS Med. 2013 Apr 23;10(4):e1001430. doi: 10.1371/journal.pmed.1001430 (PMC3637008; doi:10.1371/journal.pmed.1001430)
Supplement: Text S1 — Extended methods and results. (DOCX) [file pmed.1001430.s001.docx]

**Text S1**

*Statistical Modeling*

## In order to characterize associations between the long-term concentrations of PM_2.5_ and IMT at baseline and IMT progression, we fit a longitudinal mixed model with random slopes and intercepts for each subject in R v.2.10.1.[[21](#_ENREF_21)] This modeled examined the included three components ({predictors of baseline IMT}, [predictors of progression from baseline], and (time-varying effects)), as follows:

Y*_kit_*={α*_0_*+a*_ki_*+ X*_ki0_*α*_1_*+**Z***_ki0_***α***_2_*}+[β*_o_*+ b*_ki_*+X*_kit_*β*_1_*+**W***_kit_***β***_2_*+**R***_ki0_***β***_3_*]v*_kit_* +(**U***_kit_***γ**)+ε*_kit_*

Where for each person *i* and MESA area *k* at *t*^th^ follow-up visit :

Y*_kit_* = measured IMT

X*_kio_* = average concentration in the year prior to baseline

X*_kit_* = multi-annual average concentration between exam *t* and baseline

Z*_kio,_ U_kit_* = vectors of covariates related to baseline IMT

W*_kit_*, R*_kio_*= vectors of covariates related to IMT progression

v*_kit_* = time between the baseline and t^th^ follow-up visit

a_ki,_ b_ki_ = subject-specific random effects for baseline IMT and IMT progression

ε*_kit_* = measurement error associated with Y*_kit_*

This model captures different parameterizations of the PM_2.5_-IMT relation. First, X*_ki0_*α*_1_* characterizes the cross-sectional association between PM_2.5_ levels preceding the baseline exam and baseline IMT. X*_kit_*ν*_kit_* β*_1_* characterizes IMT progression as a function of the average concentration over follow-up. In secondary analyses, we decomposed the average concentration during follow-up into baseline concentrations (X*_kio_*v*_kit_*β*_1’_*) and the change in concentrations over follow-up ((X*_kit_-*X*_kio_)*v*_kit_* β*_1’’_*) to independently assess the associations of IMT progression with each of these two distinct exposures. We furthermore explicitly explored the relationship between change in IMT over the follow-up period as our outcome and the change in PM_2.5_ concentrations over time, controlling for time and other risk factors for change.

Although similar distributions were observed for the average concentrations between follow-up exams and at baseline (cor(X*_ki0_*, X*_kit_*) = 0.99 for between area contrasts; cor(X*_ki0_*, X*_kit_*) = 0.60 for within-area contrasts), the correlation between X_kio_ and X_kit_*v_kit_ that were used in the model were substantially lower. Standard model diagnostics were also explored including graphics of residuals for evidence of non-normality, influential outliers, and omitted covariates.

*Results for Other Estimates of Pollution*

In our primary analyses, individual-level concentrations accounting for residential history were estimated for each participant using a spatio-temporal model. To facilitate comparison to other investigations without access to the extensive monitoring data available through MESA Air, we also explored the associations between IMT and average concentrations of PM_2.5_ measured at the nearest AQS monitor and with living near a major roadway (i.e., within 100 meters of an interstate or US highway or within 50 meters of a state or county highway as defined by the US Census Feature Class Codes A1, A2, and A3). Comparable averaging periods were assumed as in the main analysis.

Table S1 illustrates qualitatively consistent findings between exposure estimates using the nearest regulatory monitoring data and our modeled concentrations. While cross-sectional associations with nearest monitor data were more robust to control for study site than associations with modeled PM_2.5_ estimates, associations with progression were weaker than with our primary exposure estimates. Living within close proximity to a major roadway was not associated with baseline IMT or IMT progression.

Table S1. Comparison of Mean Differences (95% Confidence Interval) in IMT at Baseline and IMT Progression Over Time Associated with Ambient Pollution Estimated Using the MESA Air Spatio-Temporal Model, Nearest Regulatory Monitor, and Nearness to a Major Roadway

|  | Overall  Associations | Within-City Associations |
| --- | --- | --- |
| Baseline IMT (µm) per 2.5 µg/m^3^ of Baseline PM_2.5_ | | |
| Modeled PM_2.5_ | 6.3 (2.8 to 9.8) | 0.4 (-9.1 to 9.9) |
| Nearest Regulatory PM_2.5_ | 9.2 (4.9 to 13.5) | 9.3 (0.2 to 18.5) |
| Near Roadway | -8.8 (-19.4 to 1.9) | -6.5 (-17.4 to 4.4) |
|  |  |  |
| Progression of IMT (µm/yr) per 2.5 µg/m^3^ of Average Follow-Up PM_2.5_ | | |
| Modeled PM_2.5_ | 0.4 (-0.4 to 1.2) | 5.0 (2.6 to 7.4) |
| Nearest Regulatory PM_2.5_ | 0.1 (-0.9 to 1.0) | 2.4 (-0.5 to 5.3) |
| Near Roadway | 0.4 (-1.9 to 2.7) | 1.4 (-1.0 to 3.8) |

Models controlled for age, sex, race/ethnicity, education, a neighborhood socio-economic score (derived from census tract level data on education, occupation, median home values, and median household income), adiposity (1/height, 1/height^2^, weight, waist, and 1/hip), pack-years at baseline, smoking status, HDL, total cholesterol, statin use, diabetes mellitus (using the 2003 ADA fasting criteria algorithm), systolic blood pressure, diastolic blood pressure, hypertensive diagnosis, and hypertensive medications

*Alternate Modeling Strategy*

To confirm that our findings were not overly sensitive to our model specification, we also explored the relationship between the change in IMT over the follow-up period and the change in PM_2.5_ concentrations, controlling for time and other risk factors. In this model, we found that a 1 µg/m^3^ increase in concentrations over the follow-up period was associated with a 1.9 µm (95% CI: -0.5 to 4.2 µm) and 3.4 µm (95% CI: 0.9 to 5.3 µm) larger change in IMT over follow-up, before and after control for site, respectively.
